# Complex hereditary spastic paraplegia associated with episodic visual loss caused by *ACO2* variants

**DOI:** 10.1038/s41439-021-00136-y

**Published:** 2021-01-26

**Authors:** Takenori Tozawa, Akira Nishimura, Tamaki Ueno, Akane Shikata, Yoshihiro Taura, Takeshi Yoshida, Naoko Nakagawa, Takahito Wada, Shinji Kosugi, Tomoko Uehara, Toshiki Takenouchi, Kenjiro Kosaki, Tomohiro Chiyonobu

**Affiliations:** 1 Department of Pediatrics, Graduate School of Medical Science, Kyoto Prefectural University of Medicine, Kyoto, Japan; 2 Department of Pediatrics, Ayabe City Hospital, Ayabe, Japan; 3 Department of Neonatology, Japanese Red Cross Society Kyoto Daiichi Hospital, Kyoto, Japan; 4 Department of Pediatrics, Tokai Central Hospital, Kakamigahara, Japan; 5 Kyoto Prefectural Maizuru Rehabilitation Center for Children, Maizuru, Japan; 6 Department of Pediatrics, Kyoto University Graduate School of Medicine, Kyoto, Japan; 7 Department of Medical Ethics/Medical Genetics, Kyoto University School of Public Health, Kyoto, Japan; 8 Center for Medical Genetics, Keio University School of Medicine, Tokyo, Japan; 9 Department of Pediatrics, Keio University School of Medicine, Tokyo, Japan

**Keywords:** Genetic predisposition to disease, Neurodegeneration

## Abstract

Most patients with homozygous or compound heterozygous pathogenic ACO2 variants present with muscular hypotonia features, namely, infantile cerebellar-retinal degeneration. Recently, two studies reported rare familial cases of ACO2 variants presenting as complex hereditary spastic paraplegia (HSP) with broad clinical spectra. Here, we report the case of a 20-year-old Japanese woman with complex HSP caused by compound heterozygous ACO2 variants, revealing a new phenotype of episodic visual loss during febrile illness.

The *ACO2* gene on chromosome 22 encodes the aconitase 2 (ACO2) protein in the mitochondrial matrix; ACO2 catalyzes the stereospecific isomerization of citrate to isocitrate in the tricarboxylic acid (TCA) cycle^1^. Pathogenic *ACO2* variants were first reported in eight individuals from two Arab families, and they had infantile cerebellar-retinal degeneration (ICRD, OMIM#614559)^2^. Subsequently, ~20 cases of pathogenic homozygous or compound heterozygous *ACO2* variants have been reported, including mild cases such as isolated optic atrophy (optic atrophy 9, OMIM#616289)^2–6^. Most patients initially present with muscular hypotonia, ataxia, seizures, progressive optic atrophy, retinal degeneration, and intellectual disabilities. Decreased aconitase activity in fibroblastic or lymphoblastic cells suggests that impaired energy metabolism in the TCA cycle is a major cause of symptoms in patients with pathogenic *ACO2* variants. Recently, cases from two families with pathogenic *ACO2* variants represented by early- or late-onset spastic paraplegia with intellectual disability and broad clinical spectra were reported^4,5^. Here, we describe pathogenic variants in the *ACO2* gene presenting as complex hereditary spastic paraplegia (HSP) with a new phenotype of episodic visual loss after every febrile infection and progressive optic atrophy. This is the third familial report and the first Asian patient with complex HSP caused by pathogenic *ACO2* variants.

The proband was born to nonconsanguineous healthy parents at 38 weeks gestational age after unremarkable delivery. She did not have a family history of neuromuscular disorders or motor development delay. Her birth weight was 2482 g, and her head circumference was 32 cm. Her motor development was delayed, and she could not walk independently at 1 year and 10 months because of progressive lower limb spasticity. Physiotherapy was subsequently provided, and she started walking independently at 2 years and 6 months. Her cognitive level was moderate disability (estimated development quotient: 50) at this time.

From 3 years of age, she experienced recurrent encephalopathy-like episodes, episodic visual loss, ataxia, and altered consciousness after every febrile illness episode. During febrile illness, she often accidentally hit her head on the wall because of her poor vision. Her visual loss recovered after defervescence, although the other symptoms remained for several weeks. In the acute phase, magnetic resonance imaging (MRI) of the cerebrum and the retrobulbar optic nerve and ophthalmoscopy revealed no abnormalities. The following laboratory results were normal: blood cell count; routine serum chemistry; glucose, ammonia, creatine kinase, lactate, and amino acid levels; and thyroid function. The urinary organic acid and amino acid profiles and the cerebrospinal fluid (CSF) results for cells, glucose, protein, and lactate were within normal limits. The electroencephalogram showed diffuse slow waves and focal spikes compatible with nonspecific encephalopathy; subsequently, antiepileptic drug therapy was initiated. However, despite treatment with the medications, episodic attacks repeatedly occurred after every episode of fever. Her lower limb spasticity and reflexes progressed with sustained clonus and extensor plantar responses.

At 18 years of age, she was admitted to our hospital with acute psychomotor agitation after infection. Cerebral and spinal MRI, CSF analysis, metabolic screening, and ophthalmological evaluations were performed during admission. Laboratory results showed no abnormalities, whereas cerebral MRI showed mild cerebellar vermis atrophy, and ophthalmoscopy showed bilaterally pale optic discs and suspected optic atrophy (Fig. 1A, B). These findings suggested a genetic cause for the complex HSP. Written informed consent was obtained from her parents in accordance with the Review Board and Ethics Committee of Kyoto University, and whole-exome sequencing (WES) was performed when she was 19 years old. Trio-based WES was performed using the SuperSelect XT Human All Exon v6 (Agilent Technologies, Santa Clara, CA). Captured libraries were sequenced using NovaSeq 6000 (Illumina, San Diego, CA). WES identified compound heterozygous missense variants in *ACO2*. The first variant was in exon 6 (NM_001098.2: c.776 G > A, p.Gly259Asp) and was predicted to be deleterious by SIFT (score 0; http://sift.jcvi.org/) and disease-causing by MutationTaster (prob 1; http://www.mutationtaster.org/). This variant is known to be a disease-causing mutation: rs786204828 (pathogenic)^6^. The second variant was located in exon 17 (NM_001098.2: c.2148 C > G, p.Asn716Lys) and has not been reported as a pathogenic variant; it was found to have an extremely low allele frequency (1.59 × 10^−6^) in the Genome Aggregation Database (http://gnomad.broadinstitute.org). This variant was predicted to be deleterious by SIFT (score 0.04) and disease-causing by MutationTaster (prob 0.999). Both variants were confirmed by Sanger sequencing; the p.Gly259Asp and p.Asn716Lys variants were found to be maternally and paternally inherited, respectively. The unaffected younger sister had a heterozygous p.Gly259Asp variant inherited maternally (Fig. 1C). We evaluated the pathogenicity of these two variants in accordance with the 2015 guidelines of the American College of Medical Genetics and Genomics. The c.776 G > A, p.Gly259Asp and c.2148 C > G, p.Asn716Lys variants were classified as pathogenic and likely pathogenic, respectively. At the age of 20 years, the proband had severe cognitive function (estimated intelligence quotient: ~30) and moderate visual impairment. She walked on her toes with spastic scissor gait and required a walking aid.

**Fig. 1:**
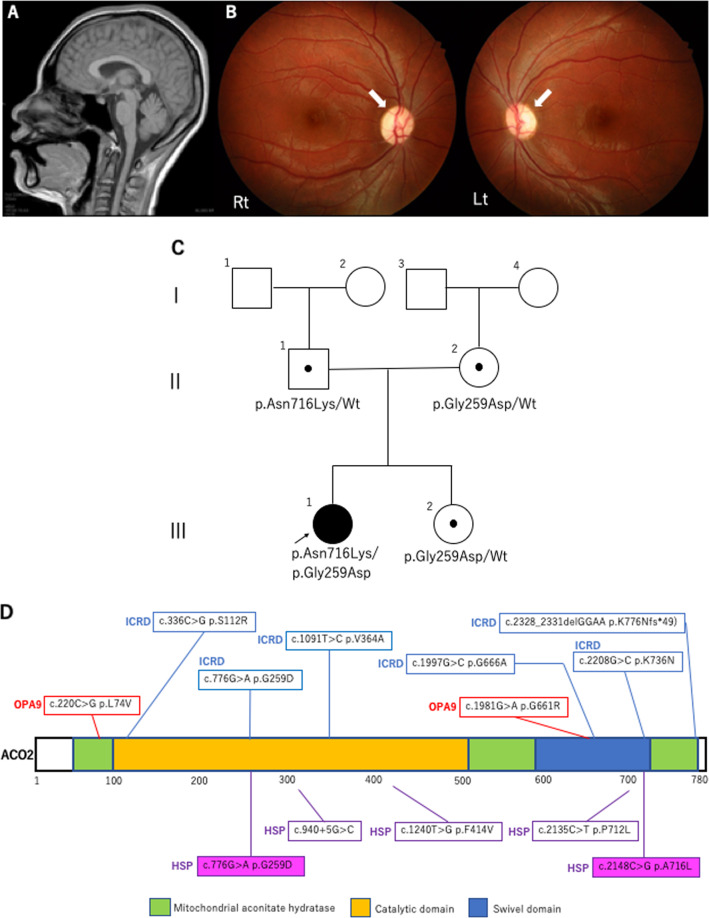
Cerebral MRI, fundus photograph and genetic information of the patient. **A** Sagittal T1-weighted MRI showed mild atrophy of the cerebellar vermis at the age of 18 years. **B** Right and left fundus photographs showed a pale optic nerve head (white arrowhead) without retinal involvement at the age of 18 years. **C** Family pedigree. Shaded symbol indicates the proband. **D** Schematic structure of the ACO2 protein. Variants in patients with ACO2-related disorders (OPA9, ICRD, and complex HSP) are shown in the diagram. OPA9: optic atrophy 9 (red box), ICRD: infantile cerebellar-retinal degeneration (blue box), HSP: hereditary spastic paraplegia (purple box), magenta-highlighted mutations: present report.

Mitochondrial ACO2 is a critical enzyme in the TCA cycle, which is the primary source of cellular metabolic energy^1^. Other TCA enzymopathies, such as deficiencies of alpha-ketoglutarate dehydrogenase, fumarase, succinate dehydrogenase, and succinyl-CoA synthase, have been previously reported to cause severe encephalopathy with muscle hypotonia, developmental delay, and retinitis pigmentosa^7–11^. The underlying pathophysiological mechanism may involve a disruption of energy metabolism and oxidative phosphorylation^7^. Therefore, aconitase deficiency is also thought to disrupt cellular energy metabolism; consistent with this, a study reported mitochondrial dysfunction in the fibroblasts of an aconitase-deficient patient^12^.

Aconitase enzymopathy is more difficult to diagnose than other TCA enzymopathies. One reason is the poor abnormalities in metabolic screening samples. An elevation of lactate levels in the blood and CSF and of specific organic acids in the urine, which is typically detected in other TCA enzymopathies, is not observed in aconitase enzymopathy. Another reason is the broad clinical spectrum of *ACO2-*related disorders, ranging from isolated optic atrophy to syndromic optic atrophy, such as ICRD that involves hypotonia, retinal degeneration, severe encephalopathy, epilepsy, and cerebellar ataxia^6^. Moreover, new phenotypes associated with spastic paraplegia were recently reported, including in this study. Table 1 shows the clinical manifestations of *ACO2*-related disorders (optic atrophy 9, ICRD, and complex HSP)^4–6,13^. Most patients with *ACO2*-related disorders have optic nerve involvement, which might be a hallmark feature of aconitase enzymopathy, but not all patients have optic atrophy^4,12^. Thus, biochemical testing and clinical phenotypes are insufficient for diagnosing aconitase enzymopathy, thereby indicating the importance of WES.

**Table 1:** Clinical manifestations of *ACO2*-related disorders.

References	Metodiev et al.					Srivastava et al.	Marelli et al.	Bouwkamp et al.		This study
Clinical presentation	OPA9		ICRD				Complex HSP			
Sex	Male	Male	Male	Male	Female	Male	Female	Male	Female	Female
Age at report (years)	36	41	Died 57 days	Died 61 days	4	18	56	28	14	20
Ethnicity	French	French	Algerian	Algerian	N/R	N/R	N/R	Arab Bedouin	Arab Bedouin	Japanese
Infantile cerebellar-retinal degeneration	−	−	+	+	+	+	−	−	−	−
Optic atrophy	+	+	Edema of optic disks	+	+	+	+	−	−	+
Spastic paraplegia	−	−	−	−	−	−	+ (Late onset)	+ (Early onset)	+ (Early onset)	+ (Early onset)
Intellectual disability	−	−	N/A	N/A	+	Severe	Mild	Severe	Mild	Severe
Seizure	−	−	N/R	N/R	+	+	−	+	−	+
Episodic visual loss	−	−	N/A	N/A	−	−	−	−	−	+
Episodic ataxia	−	−	N/A	N/A	−	+	−	−	+	+
Episodic behavioral change	−	−	N/A	N/A	−	−	−	−	+	+
MRI finding	No abnormality	No abnormality	Moderate atrophy of the cerebellum	Moderate atrophy of the cerebellum	Moderate atrophy of the cerebellum	Mild atrophy of the cerebellum	Mild atrophy of the cerebellar vermis	Mild atrophy of the cerebellum	Signal abnormality in subcortical white matter	Mild atrophy of the cerebellar vermis
Mutation	p.Leu74Val/ p.Gly661Arg	p.Leu74Val/ p.Gly661Arg	p.Gly259Asp/p.Gly259Asp	p.Gly259Asp/p.Gly259Asp	p.Lys736Asn/p.Lys776Asnfs*49	p.Val364Ala/c.2328_2331delGGAAfs	p.Pro712Leu/ c.940 + 5 G > C	p.Phe414Val/p.Phe414Val	p.Phe414Val/p.Phe414Val	p.Gly259Asp/p.Asn716Lys
Enzyme activity (%)	60	66	<5	N/P	30	N/P	50	~20	~20	N/P

*+* present, *−* absent, *MRI* magnetic resonance imaging, *N/R* not reported, *N/A* not available, *N/P* not performed, *OPA9* optic atrophy 9, *ICRD* infantile cerebellar-retinal degeneration, *HSP* hereditary spastic paraplegia.

Figure 1D shows the structure of the ACO2 protein and reported variants in *ACO2*-related disorders, including in this study. There is no hot-spot region in any *ACO2*-related disorder, and there seems to be poor genotype–phenotype correlation. Compared to previously reported *ACO2*-related disorders, the novel characteristic phenotype in the present patient was episodic visual loss during febrile infection (Table 1). The phenotypes in the present patient indicated that ACO2 plays a crucial role in energy production in the optic nerve and retina, which are highly energy-dependent structures^14^. Previous findings suggested that phenotype variation and severity depend on residual aconitase enzymatic activity^12^. Metodiev et al. reported a very severe case involving homozygous Gly259Asp variants^6^. The patient presented with syndromic optic neuropathy along with encephalopathy and cerebellar atrophy and died at 57 days because of central apnea^6^. The aconitase enzymatic activity in the patient’s fibroblasts was extremely low (~5%)^6^. However, a case report of a mild phenotype despite a marked reduction in aconitase enzyme activity^12^ suggested poor genotype–phenotype correlations in *ACO2* variants and the coexistence of genomic modifiers.

HSP is not a single disease; rather, it is a mixture of genetically heterogeneous conditions resulting in broadly overlapping clinical phenotypes. Using single-gene direct sequencing and next-generation sequencing technologies, various HSP-related gene variants have been identified^15^. These genes encode proteins with diverse molecular functions, axonal transport, specific lipid metabolism, synaptic formation, axon development, and mitochondrial function^15^. In addition to *ACO2* variants, several other HSP-associated gene variants, such as those in *PGN*, *HSPD1*, *DDHD1*, *REEP1*, and *MT-ATP6*, have been found to impair mitochondrial function^16–20^. Further reports on the causative genes of HSP would improve the understanding of the crucial role of mitochondrial dysfunction in HSP pathogenesis.

In conclusion, this case represents the third report of HSP caused by pathogenic *ACO2* variants. Although most patients with *ACO2*-related disorders present with muscular hypotonia features, it should be recognized that pathogenic *ACO2* variants comprise one of the causes of complex HSP. Patients with *ACO2*-related disorders should be evaluated for signs such as early-onset spastic paraplegia, especially those with episodic visual loss after febrile infection and progressive optic atrophy. The identification of pathogenic *ACO2* variants in patients with HSP could contribute to the development of specific therapies against HSP caused by mitochondrial dysfunction.

## Data Availability

The relevant data from this Data Report are hosted at the Human Genome Variation Database at 10.6084/m9.figshare.hgv.2951 and 10.6084/m9.figshare.hgv.2954.
